# Tumor-associated macrophages are associated with response to neoadjuvant chemotherapy and poor outcomes in patients with triple-negative breast cancer

**DOI:** 10.7150/jca.47566

**Published:** 2021-03-15

**Authors:** Jia-hui Ye, Xiao-hua Wang, Jia-jun Shi, Xi Yin, Cheng Chen, Yan Chen, Hong-Yan Wu, Shi Jiong, Qi sun, Meng Zhang, Xian-biao Shi, Guo-ren Zhou, Shahzeb Hassan, Ji-feng Feng, Xin-yun Xu, Wei-jie Zhang

**Affiliations:** 1 Dept. of General Surgery, Affiliated Drum Tower Hospital of Nanjing University Medical School, Nanjing, China.; 2 Dept. of Medical Oncology, Jiangsu Cancer Hospital Affiliated to Nanjing Medical University, Nanjing, China.; 3 Dept. of Radiotherapy, Jiangsu Cancer Hospital Affiliated to Nanjing Medical University, Nanjing, China.; 4 Dept. of Pathology, Jiangsu Cancer Hospital Affiliated to Nanjing Medical University, Nanjing, China.; 5 Dept. of Pathology, Affiliated Drum tower Hospital of Nanjing University Medical School, Nanjing, China.; 6 Dept. of Breast Surgery, Jiangsu Cancer Hospital Affiliated to Nanjing Medical University, Nanjing, China.; 7 Northwestern University Feinberg School of Medicine, Chicago 60611, IL, United States.

**Keywords:** triple-negative breast cancer, tumor-associated macrophages, neoadjuvant chemotherapy, prognosis

## Abstract

**Background and objective:** Tumor-associated macrophages (TAMs) play an essential role in tumor progression and metastasis. However, the role of TAMs in neoadjuvant chemotherapy (NAC) is unclear and need to be identified. The main subject of this study was to investigate whether TAMs are related to the chemotherapeutic response with triple-negative breast cancers (TNBC).

**Methods:** We retrospectively analyzed pretreatment tissue from patients who received NAC and followed by a mastectomy or breast-conservation for stage II-III TNBC in this study. The association between TAMs and the pathological complete response (pCR) rate of TNBC to NAC was analyzed. In addition, the correlation of the TAMs with recurrence-free survival (RFS) in patients with TNBC was also evaluated.

**Results:** Of the 91 patients, 31 (34.1%) patients experienced pathological complete response (pCR) after completion of NAC. Regarding the chemotheraptic response, patients with low infiltration of CD163^+^ macrophages achieved a significantly higher rate of pCR. Importantly, Kaplan-Meier survival shown that patients with high infiltration of CD163^+^ macrophages and non-pCR had poor OS and RFS.

**Conclusions:** our data showed that TAMs may predict chemotherapeutic response and can be used as a promising prognostic candidate for poor survival in TNBC patients treated with NAC.

## Introduction

Triple-negative breast cancer (TNBC), which represents 15%-20% of all invasive breast neoplasms, is a subtype of breast cancer characterized by negative expression of oestrogen and progesterone receptors, as well as lack of HER2 amplification 1.

TNBCs are usually composed of histologically high-grade and biologically aggressive cancer cells and tend to recur within three years of diagnosis with poor prognosis 2. Currently, neoadjuvant chemotherapy (NAC) followed by definitive operation is the standard therapy for locally advanced TNBC. It is also an alternative treatment for TNBC of early stage, improving the opportunity for breast-conserving surgery (BCS) 3.

The usage of NAC can also provide insight into biological features and differential responses regarding the treatment of tumors. The effect of NAC, usually assessed by pathological responses of surgical samples, has significantly influenced patient outcomes. Patients who experienced pathological complete response (pCR) have a relatively lower risk of recurrence or death compared to those who did not achieve pCR after NAC 4, 5.

Tumor microenvironment (TME) plays a principal role in oncogenesis and cancer progression. Accumulating research suggests that the high infiltration of tumor-associated macrophages (TAMs) is a critical player in tumor growth, angiogenesis, and treatment resistance. Increasing evidence shows that TAMs are a poor prognostic factor for TNBC patients 6-9.

However, few studies have investigated the association between infiltration of TAMs and response to NAC in TNBC. Therefore, in the present study, we aimed to investigate whether or not high infiltration of TAMs is less likely to respond to NAC, in terms of pCR for TNBC patients in the neoadjuvant setting.

## Patients and Methods

### Patients and clinical data selection

In this study, a total of 91 primary TNBC patients, ranging from 23 to 65 years were included. Patients had locally advanced, non-resectable tumors, not suitable for breast-conservation, and were treated with NAC and either mastectomy or BCS at Drum Tower Hospital and Jiangsu Cancer Hospital. Inclusion criteria were being female and having primary invasive TNBC that was in stages II to III. Patients with male breast cancer, carcinoma in situ, stage IV breast cancer, bilateral breast cancer, inflammatory breast cancer and those who received any therapy (chemotherapy, endocrine therapy, and radiotherapy) before NAC were all excluded from this study.

Before NAC, TNBC was confirmed histologically by core needle biopsy with immunohistochemistry. Palpable or ultrasound-detected lymph nodes were biopsied with fine-needle aspiration at the time of diagnosis. Initial staging was determined by physical examination, blood chemistry, ultrasonography, bone scintigraphy, and computed tomography (CT), which helped evaluate organ function and exclude distant metastasis.

### Pathology and immunohistochemistry

Two experienced pathologists who were blinded to the corresponding clinical and pathological data assessed the results under the guidance of 2012 WHO classification of tumors of the breast 10. Using pre-NAC core needle biopsy tissue, specimens were histologically graded by the Nottingham grading system 11, 12.

TAMs density was confirmed by CD163 expression and further examined (per mm^2^) under a light microscope (Carl Zeiss Axio Scope. A1 microscope) at 400× magnification in five fields per case. Immunohistochemical staining was performed with the mouse monoclonal anti-CD163 antibody (Abcam, Cambridge, MA, USA) under the manufacturer's instructions and using a 3-3'-diamino-benzidine (DAB) Substrate Kit (Maxin, Jiehao Co., Ltd, Shanghai, China), on the invasive component of core needle biopsy specimens. Positive and negative controls were included in the experimental setup. Normal mouse serum (instead of the primary antibody) served as the negative control. A non-specific staining blocking agent (Dako, Kyoto, Japan) was also used to prevent non-specific binding. The mean number of positive cells was determined for each case. Low density (infiltration) was identified based on the number of positive cells lower than the average level; otherwise, it was considered a high density (infiltration).

All core-needle biopsy samples and surgical specimens were assessed by two experienced pathologists (S.Q. and S.J.) who were blinded to the clinical parameters. When inconsistencies were more than 5%, reevaluation was conducted for consensus.

### Treatment and chemotherapy response

Among the 91 patients who received NAC, all the patients received 4 to 8 cycles of NAC and the treatment consisted of anthracycline- and taxane-based regimens, including TEC, EC-T and PE. Following NAC, operations (mastectomy or BCS) were performed to remove the primary tumor and axillary sentinel lymph node biopsy or axillary lymph node dissection were conducted to excise the lymph nodes.

Indications for postoperative radiotherapy included all cases that had breast-conserving therapy and also consisted of some cases with initial tumor size greater than 5 cm or those with clinical N2 status that underwent mastectomy. Patients received postoperative adjuvant chemotherapy therapy based on their clinical and pathological factors after operation.

Pathological examination included histology type, tumor extension and lymph node and resection margin. The clinical responses to NAC were estimated in accordance with the response evaluation criteria in solid tumors (RECIST) version 1.1. and were based on Nuclear Magnetic Resonance Imaging (MRI) and ultrasonic examinations. The definition of pathologic complete response (pCR) was the lack of all signs of invasive cancer in breast tissue and lymph nodes of the resected sample that were removed during surgery after NAC; carcinoma in situ in breast residuals were allowed (ypT0/is ypN0).

### Ethics statement

This retrospective study was approved by the clinical ethical committee of both hospitals (Drum Tower Hospital Ethical Committee and Jiangsu Cancer Hospital Ethical Committee), and all participants or relatives gave their informed consent.

### Statistical analysis

The associations between CD163 expression status, clinicopathologic features, and pathological response to NAC were analyzed by Pearson's chi-square test or Fisher's exact test. A multivariate logistic hazard analysis was used for analyzing whether a factor was an independent predictor of pCR by the Cox regression model. Survival curves were estimated using the Kaplan-Meier method, and the log-rank test was used to evaluate and compare recurrence-free survival (RFS) by CD163 and response to NAC. The statistical analysis was performed with SPSS (SPSS, Chicago, IL) software for Windows, version 17.0. The *P* value less than 0.05 was considered statistically significant.

## Results

### Patient characteristics

A total of 91 cases were enrolled in this retrospective study and the detailed clinical and pathological features of TNBC patients with NAC are presented in Table 1. Most of the patients were over 35 years old, and more than half were post-menopausal. Predominant histology was invasive ductal carcinoma (77.0%) and the majority of patients were lymph node positive at pre-NAC diagnosis (66.8%). The pre-NAC clinical T stage of most of the cases was cT2, accounting for 73.6% of the patients.

More than two thirds of cases expressed a high Ki67 proliferation index (≥14) and only 38.5% of cancers were well/moderately differentiated (G1-G2). Following NAC, 34 cases (37.4%) received BCS approach, while the remaining 57 (62.6%) cases underwent mastectomy. Overall, pCR was observed in 31 patients (34.1%), including one patient with residual ductal carcinoma.

### Relationship between infiltration of TAMs and clinicopathological characteristics of patients

**Figure 1:**
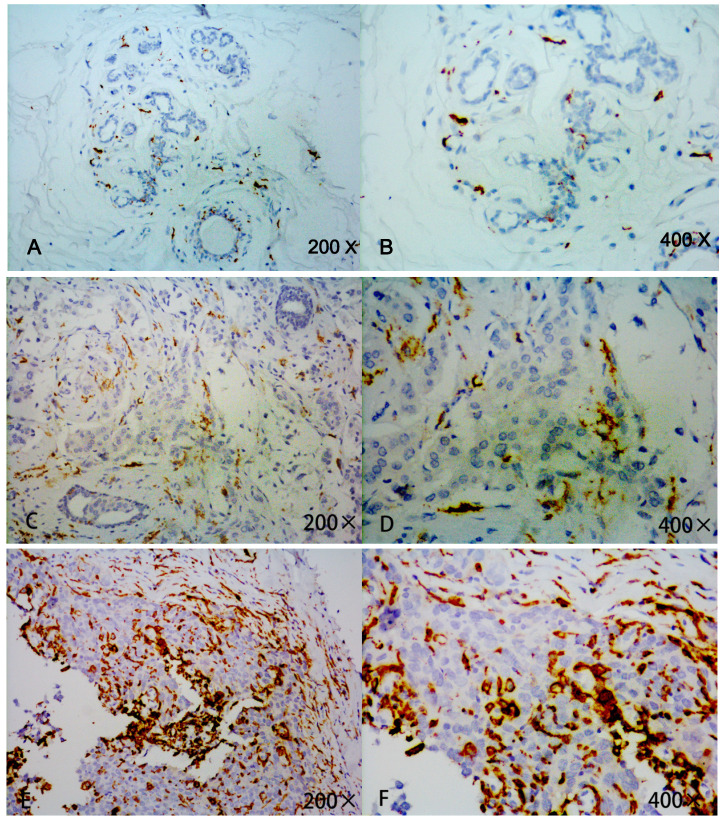
Detection of CD163 expression in breast cancer tissue and adjacent normal tissue by immunohistochemistry. Strong CD163 immunoreactivity was identified in aggressive cancer (E, F), weak immunoreactivitywas identified in low or moderate cancer(C, D) and barely seen in normal tissue (A, B).

In this cohort of 91 patients, high expression of CD163 was detected in 47.3% (43/91) cases of TNBC. No significant differences were found between high expression of CD163 and low expression of CD163 in regards to age, tumor size, menstrual status, Ki-67 expression, and surgical method (Table 1). However, high infiltration of TAMs was closely associated with aggressive behaviors, such as advanced stage, nodal metastasis, lymphovascular invasion, etc. (Figure 1).

### Response to neoadjuvant chemotherapy in patients with high and low infiltration of TAMs

In the present study, a total of 31 cases (34.1%) achieved a pCR after NAC. The correlation between the pCR and different clinical-pathological features was demonstrated in Table 2, pCR was significantly related to tumor size (*P* =0.036), CD163 expression (*P* =0.025), LN status before NAC (*P* = 0.029) and LVI (*P* = 0.027). There was no significant association with age, menstrual status, Ki67 index, histologic grade and type between the two groups.

According to the infiltrative density of TAMs (identified by CD163 expression), we divided all the TNBC patients into two groups (high *vs.* low) (Figure 1). Compared with patients having low infiltration of TAMs, those with high infiltration of TAMs experienced lower pCR rate after therapy (42.9% vs. 20.0%, *P* = 0.025). In multivariate logistic analysis, low expression of CD163 was determined as an independent significant predictive factor for pCR (OR: 3.333; *P* = 0.031; Table 3).

### Survival and prognostic factors for recurrence-free survival in LABC who received NCT

The median follow-up time of all patients was 45.8 months and the OS of all the patients was 61.02 ± 18.57 months. 20 patients died (14 because of tumor recurrence and 6 due to other illness without relapse), and the remaining 71 patients were alive (including 3 recurrences) when follow-up ended. A total of 17 patients (18.7%) developed recurrences. 16 of the 65 non-pCR cases relapsed, including local recurrence and distant metastasis. However, only one of the patients with pCR had recurrence with lung metastasis.

**Figure 2:**
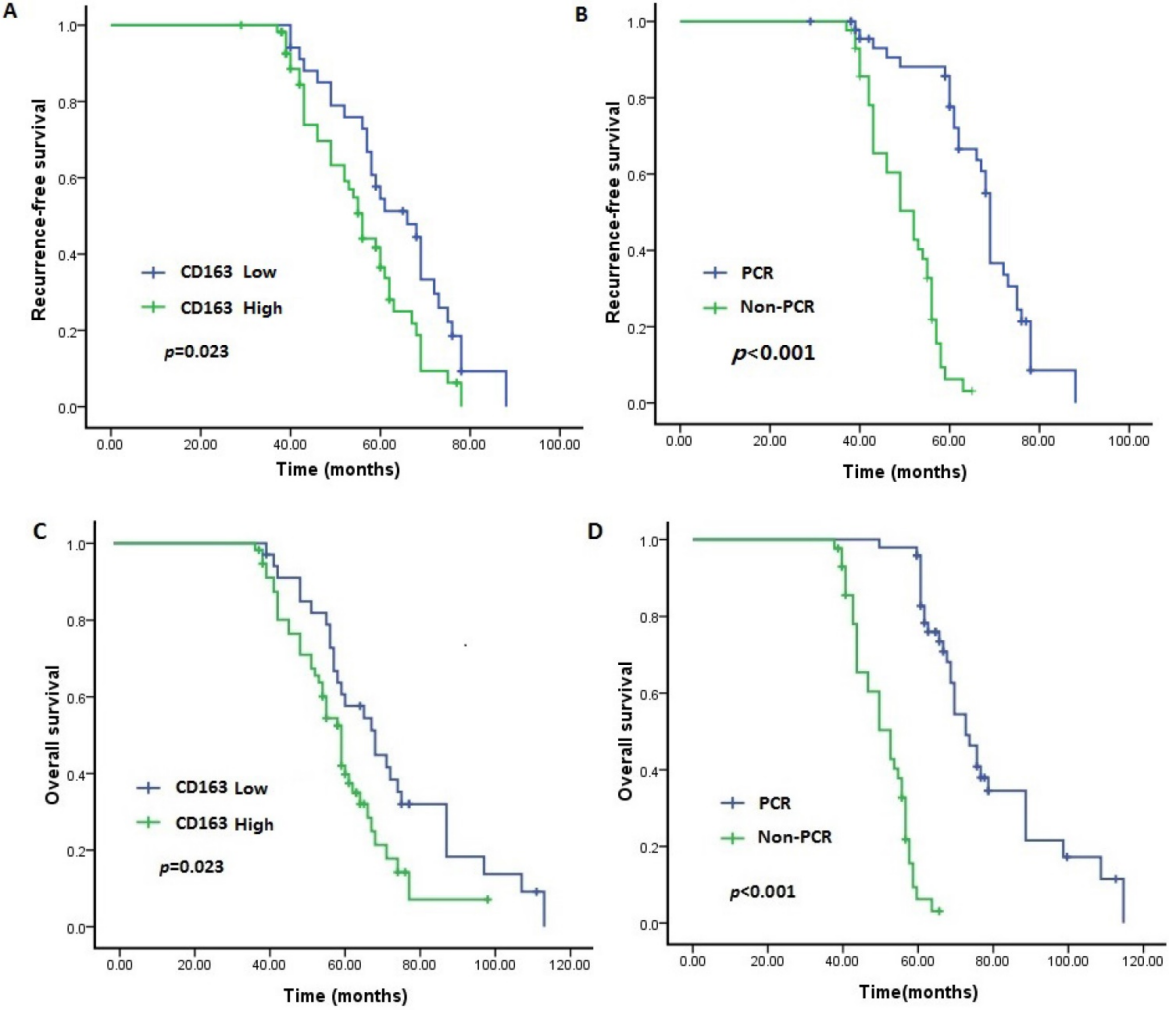
Expression of CD163 and pCR status predict outcome of TNBC. Patients with low level of CD163 demonstrated longer RFS and OS than those with high level (*P*<0.05). Patients who achieved pCR had a better RFS and OS than those with non-pCR (*P*<0.05). A. RFS between patients with low and high level of CD163; B. RFS between patients with pCR and Non-pCR. C. OS between patients with low and high level of CD163; B. OS between patients with pCR and Non-pCR. TNBC: triple negative breast cancer; RFS: recurrence-free survival; OS: overall survival.

In the survival analysis, as demonstrated in the Kaplan-Meier survival curves (Figure 2A-D), the OS and RFS rates were significantly lower for patients with high infiltration of TAMs than for those with low infiltration (*P* = 0.023 and *P* = 0.013, respectively). Moreover, as expected, the OS and RFS rates were significantly different between the pCR and non-pCR groups, and failure to achieve a pCR was related to worse long-term outcomes (both *P* < 0.001).

## Discussion

TNBC is an aggressive breast cancer characterized by poorly differentiated histologies, high rates of metastatic spread and poor prognosis, with chemotherapy still remaining the backbone of therapy 4, 13. Despite the dismal outcomes of TNBC, research has shown that TNBC is more sensitive to chemotherapy drugs than other molecular subtypes 14-17.

In the neoadjuvant setting, TNBC has a relatively high tendency for achieving pCR; however, whether or not pCR following neoadjuvant chemotherapy can be a predictor for improvement in overall survival has conflicting data. Carey et al. show that the superiority of pCR is not explicitly translated into an improvement in OS because of poor outcomes of non-pCR responders 18. On the contrary, the results of Fisher et al. reported a favorable OS in patients with pCR following NAC compared with patients receiving adjuvant therapy 4. Although previous clinical studies have not suggested a survival difference between patients receiving neoadjuvant versus adjuvant chemotherapy for breast cancer, our study demonstrates an RFS and OS benefit in patients with pCR following NAC compared with patients with non-pCR.

However, standard neoadjuvant therapy results in pCR rates of slightly over 30% and 19, 20, as for early-stage TNBC patients, the pCR rate ranges from 49% to 71% when pembrolizumab is combined with chemotherapy as a neoadjuvant treatment 21. The results of our study are consistent with the data of previous studies, showing that the overall pCR rate of TBNC patients is 34.1%, and for low CD163 expression cases, the pCR rate is 42.9%, which is more than twice the pCR rate of high CD163 expression patients. The response prediction is extremely important for NAC candidates. Our findings show that loss of CD163 expression has an important predictive value and is related to the possibility of better response to neoadjuvant chemotherapy in locally advanced TNBC.

Tumor microenvironmental factors contribute to tumorigenesis, metastasis and relapse following therapy, and could be conducive to a better understanding of NAC response. Immunity can induce the potential of anti-tumor effectiveness of selected chemotherapeutic agents, but the molecular mechanisms remain to be clarified. Recent studies demonstrate that TAMs, the predominant immune-related stromal cells in TME, play an essential role in promoting cancer progression, and high infiltration of TAMs in primary tumors is associated with poor prognosis in TNBC patients 8, 9, 22. In breast cancer, patients with high amounts of TAMs and low amounts of cytotoxic T cells had a limited response to NAC, and blocking signals, which mediate macrophage recruitment plus chemotherapy, significantly decreased primary cancer progression, reducing metastasis 23. Yamamoto et al. demonstrated that high infiltration of CD163+ or CD206+ macrophages was significantly associated with poor pathological response to NAC, and esophageal cancer patients with high infiltration of M2 macrophages exhibited poor prognosis compared to those without high infiltration by using pretreatment biopsy sample 24.

In this study, we selected 91 patients who underwent NAC in order to identify whether or not TAMs infiltration was connected with response to NAC and could predict prognosis for TNBC patients who underwent NAC. The results of the present study showed that high infiltration of TAMs was related to a poor response to chemotherapy and dismal outcomes for TNBC patients who underwent NAC followed by operation. The results from the current study suggest that CD163+TAMs account for 47.3% of all TNBC patients (43/91, 47.3%). It also shows that high infiltration of CD163+TAMs is closely associated with aggressive behaviors, such as advanced stage and lymphovascular invasion, and could predict poor response to NAC in patients with TNBC. Our present study also shows the significant over-expression of CD163 in TNBC with lymph nodal metastasis. This finding can be very helpful, as it is important to recognize clinical and pathological factors that may influence lymph node metastasis. Survivcd1al analysis confirmed that patients with high infiltration of polarized TAMs have poor OS and RFS.

However, the relationship between the infiltration of TAMs and chemo-resistance of NAC in breast cancer has not been fully clarified. Jinushi et al. 25 suggested that TAMs produce large amounts of milk-fat globule epidermal growth factor-8 (MFG-E8), and that TAM-specific MFG-E8 nurtures cancer stem cells and mediates drug resistance through activating STAT3 and Hedgehog signals. The anti-tumor chemotherapy resistance mediated by MFG-E8 is enhanced through the interplay between MFG-E8 and IL-6 released from TAMs. Another study by Zhang et al. 26 reported that chemokines, such as CSF-1 and SDF-1α, which were released from tumors during chemotherapy, increased the amount of TAMs in the tumor and resulted in chemoresistance. They also found that depletion of TAMs enhances the effects of sorafenib in metastatic liver cancer models. As for breast cancer, NAC increases M2 macrophage infiltration, and M2 macrophages enhance cyclopamine resistance and protect breast cancer cells from the antitumor effects of the Hh pathway inhibitor by secreting IL6 27. Further studies are needed to investigate the detailed mechanisms on how TAMs modify the drug resistance in NAC. In the current study, infiltration of TAMs was much more prominent in TNBC that had advanced tumor stage before NAC, a high histological grade, and a poor response to chemotherapy when compared with those with early tumor stage before NAC, a low histological grade and a good response to chemotherapy. The evidence may suggest that TAMs play a key role in chemotherapeutic resistance for patients with TNBC.

Our article has some limitations. First, the size of sample is relatively small. Given the heterogeneous distribution of immune cells, tumor tissue specimens obtained by core-needle biopsy from the neoplasm may be not necessarily representing the whole tumor microenvironment 28. Second, polarized TAMs defined by CD163 was limited, the macrophages demonstrate high plasticity under kinds of stimulus 29, however till now there aren't any reliable surface markers to identify and evaluate TAMs. Finally, previously independent cohort of breast cancer patients shown that residual cancer burden was prognostic for long-term survival after NAC in all three phenotypic subsets of breast cancer 30, and taking the aforementioned limitations together with a small sample size suggest that our findings need to be validated with prospective large-scale independent cohorts clinical studies, and some basic researches should be conducted in the near future.

In conclusion, high infiltration of TAMs was related to the chemotherapeutic response in the NAC setting and was also associated with aggressive behaviors can be used as a promising prognostic candidate for poor survival in TNBC patients treated with NAC.

## Figures and Tables

**Table 1:** The relationship between expressions of CD163 and clinicopathologic features of TNBC patients

	n (%)	High CD163 (%)	Low CD163 (%)	χ^2^-value	*P*
**Age (yr)**				0.614	0.432
≤35	20 (22.0)	11 (52.4)	9 (47.6)		
>35	71 (78.0)	32 (45.7)	39 (54.3)		
**Menstrual status**				0.367	0.544
Pre-menopause	39 (42.9)	17 (43.6)	22 (56.4)		
Post-menopause	52 (57.1)	26 (50.0)	26 (50.0)		
**Tumor Stage before NAC**				4.586	**0.032**
cT1-cT2	57 (62.6)	22 (38.6)	35 (61.4)		
cT3-cT4	34 (37.4)	21 (61.8)	13 (38.2)		
**Histological grade**				4.357	**0.037**
1/2	35 (38.5)	12 (34.3)	23 (65.3)		
3	56 (61.5)	31 (55.4)	25 (44.6)		
**Histological type**				0.265	0.607
Ductal	78 (85.7)	36 (46.2)	42 (53.8)		
Others	13 (14.3)	7 (53.8)	6 (46.2)		
**LVI**				4.612	**0.032**
Negative	53 (58.2)	20 (37.7)	33 (62.3)		
Positive	38 (41.8)	23 (60.5)	15 (39.5)		
**Nodal status before NAC**				5.968	**0.015**
Negative	33 (36.3)	10 (30.3)	23 (69.7)		
Positive	58 (63.7)	33 (56.9)	25 (43.1)		
**Ki67**				3.705	0.054
<14	28 (30.8)	9 (32.1)	19 (67.9)		
≥14	63 (69.2)	34 (54.0)	29 (46.0)		
**NAC regimen**				0.625	0.732
EC-T	19 (20.9)	10 (52.6)	9 (47.4)		
TEC	59 (64.8)	28 (49.2)	31 (50.8)		
PE	13 (14.3)	5 (38.5)	8 (61.5)		
**pCR with NAC**				8.676	**0.003**
Yes	31 (34.1)	8 (25.8)	23 (74.2)		
No	60 (75.9)	35 (58.3)	25 (41.7)		
**Surgery**				0.214	0.644
Mastectomy	57 (62.6)	28 (49.1)	29 (50.9)		
BCS	34 (37.4)	15 (44.1)	19 (55.9)		

NAC: neoadjuvant chemotherapy; LVI: lymphovascular invasion; EC-T: epirubicin, cyclophosphamide, docetaxel; TEC: docetaxel, cyclophosphamide, epirubicin PE: paclitaxel, epirubicin; pCR: pathological complete response; BCS: breast-conserving surgery.

**Table 2:** The correlation between the pCR and different clinical-pathological features

Variable	n (%)	pCR (%)	χ^2^-value	*P* value
**Age (yr)**			0.402	0.526
≤35	20 (22.0)	8 (40.0)		
>35	71 (78.0)	23 (32.4)		
**Menstrual status**			0.016	0.898
Pre-menopause	39 (42.9)	13 (33.3)		
Post-menopause	52 (57.1)	18 (34.6)		
**Tumor size before NAC**			4.390	0.036
cT1-cT2	57 (62.6)	24 (42.1)		
cT3-cT4	34 (37.4)	7 (20.6)		
**Histological grade**			3.436	0.061
1/2	35 (38.5)	17 (37.1)		
3	56 (61.5)	14 (23.2)		
**Histological type**			0.073	0.786
Ductal	78 (85.7)	27 (34.6)		
Others	13 (14.3)	4 (30.8)		
**LVI**			4.919	0.027
Negative	53 (58.2)	23 (43.4)		
Positive	38 (41.8)	8 (21.1)		
**Nodal status before NAC**			4.793	0.029
Negative	33 (36.3)	17 (51.5)		
Positive	58 (63.7)	15 (24.1)		
**Ki67**			2.752	0.097
<14	28 (30.8)	13 (46.4)		
≥14	63 (69.2)	18 (28.6)		
**CD163 expression**			5.010	0.025
High	35 (38.5)	7 (20.0)		
Low	56 (61.5)	24 (42.9)		
**NAC regimen**			0.268	0.875
EC-T	19 (20.9)	7 (31.6)		
TEC	59 (64.8)	19 (25.4)		
PE	13 (14.3)	5 (30.8)		

NAC: neoadjuvant chemotherapy; LVI: lymphovascular invasion; EC-T: epirubicin, cyclophosphamide, docetaxel; TEC: docetaxel, cyclophosphamide, epirubicin PE: paclitaxel, epirubicin; OR:Odds ratio; pCR: pathological complete response; BCS: breast-conserving surgery; CI: Confidence interval.

**Table 3:** Multivariate logistic analysis of factors influencing pCR in TNBC patients underwent neoadjuvant chemotherapy

Variables	OR	95% CI	*P*-value
Tumor size before NAC (cT1-cT2 vs cT3-cT4)	4.375	1.353-14.151	0.014
LNS before NAC (N0-N1 vs N2-N3)	0.636	0.248-1.631	0.347
LVI status (Negative vs Positive)	2.545	0.949-6.830	0.064
TAM infiltration (Low vs High)	3.333	1.117-9.946	0.031

TAM: tumor-associated macrophages; LNS: lymph node status; LVI: lymphovascular invasion; OR:Odds ratio; CI: confidence interval.
